# Supplementary figures and images for: The ribosome-inactivating proteins MAP30 and Momordin inhibit SARS-CoV-2
Source: PLoS One. 2023 Jun 29;18(6):e0286370. doi: 10.1371/journal.pone.0286370 (PMC10310010; doi:10.1371/journal.pone.0286370)

**A**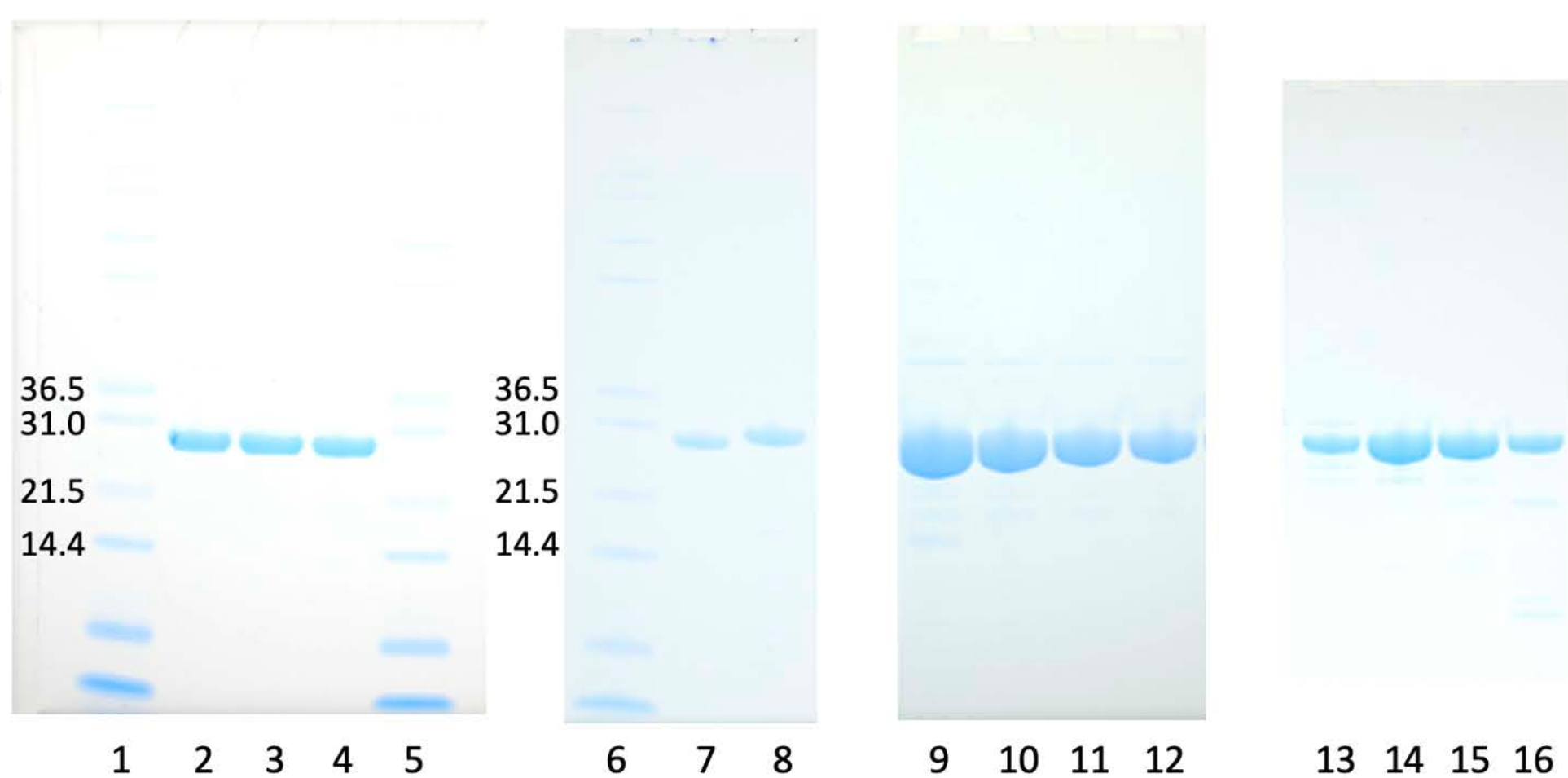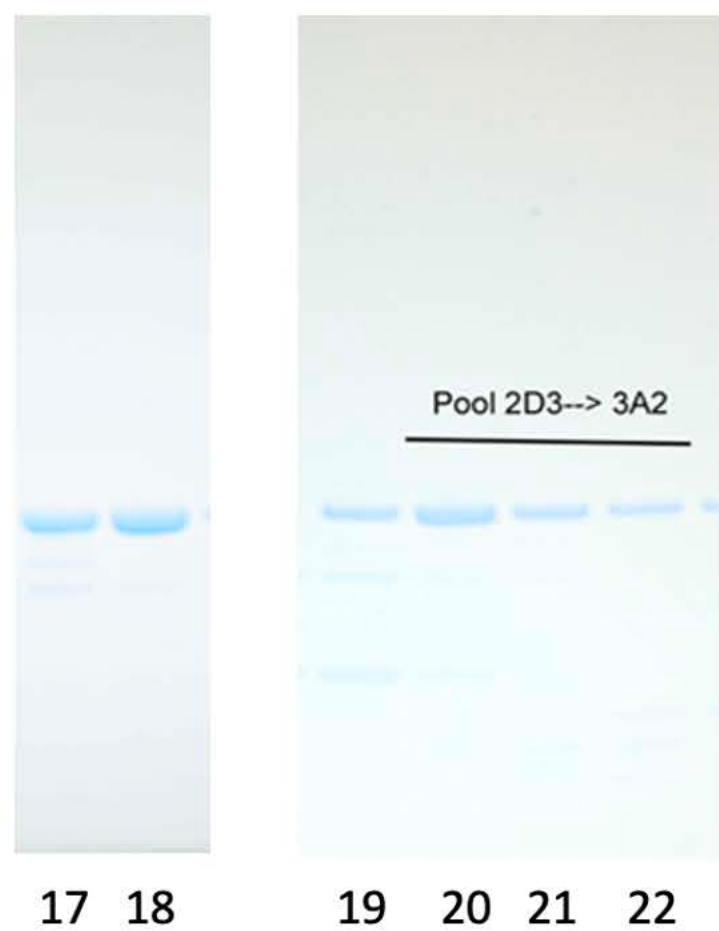**B**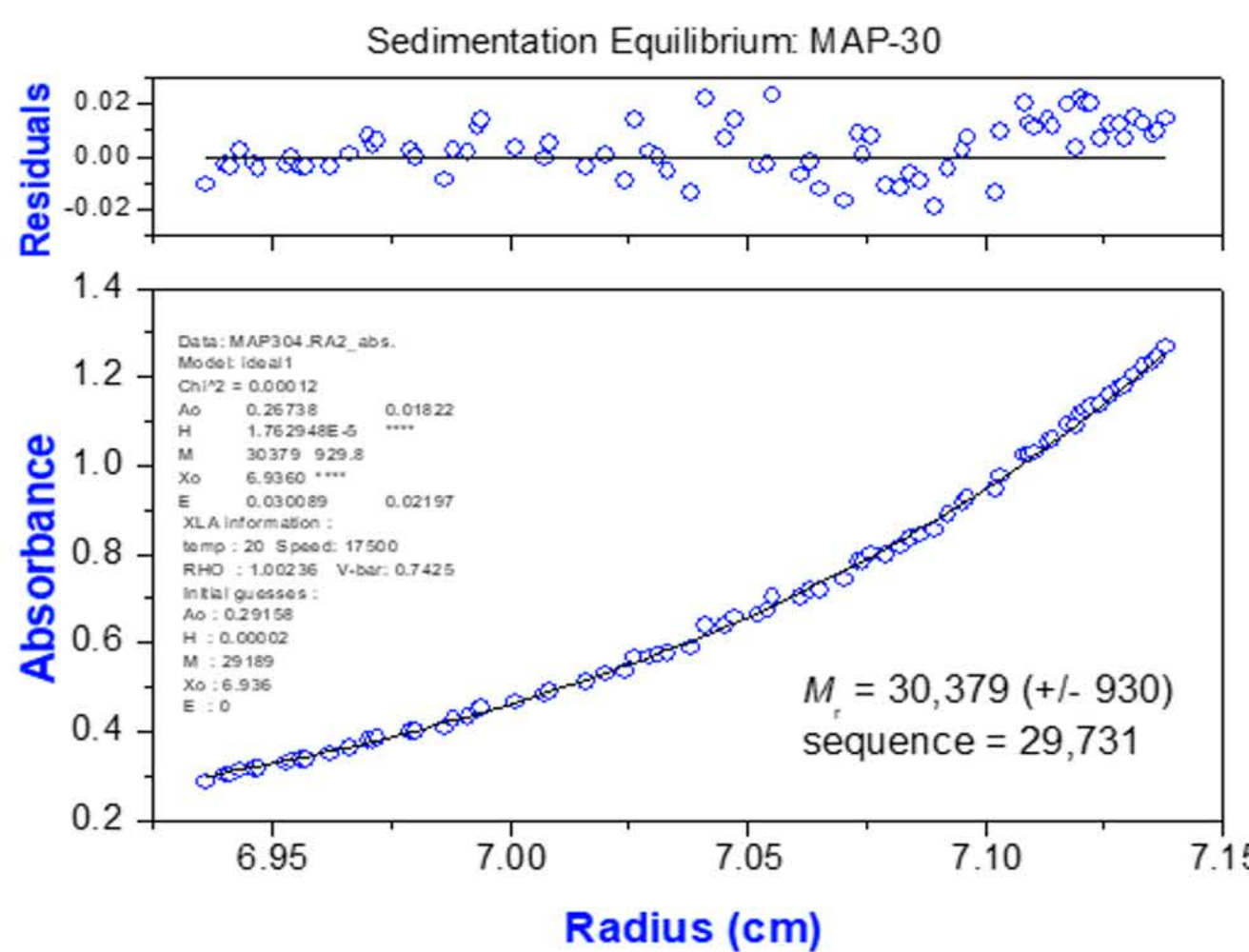**C**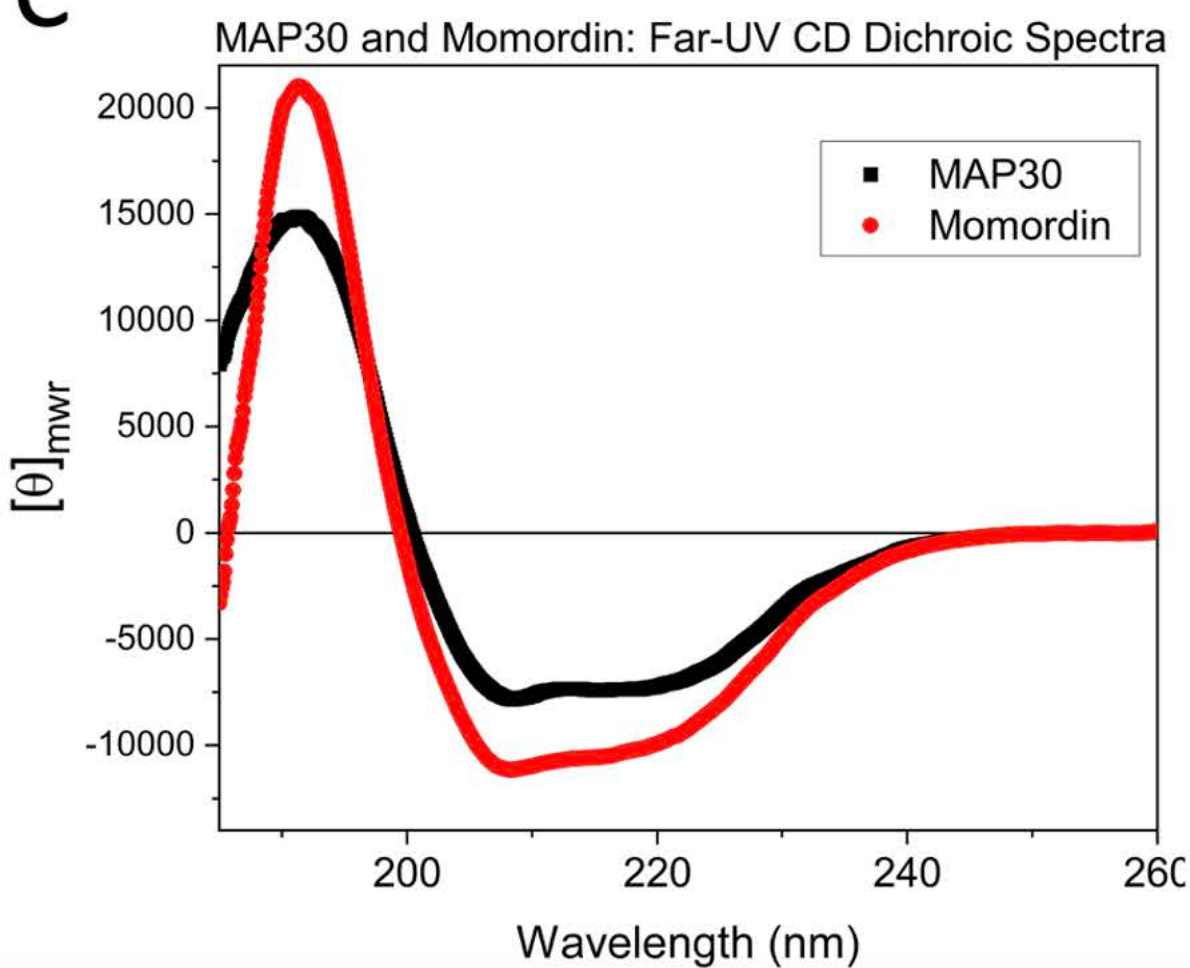**D**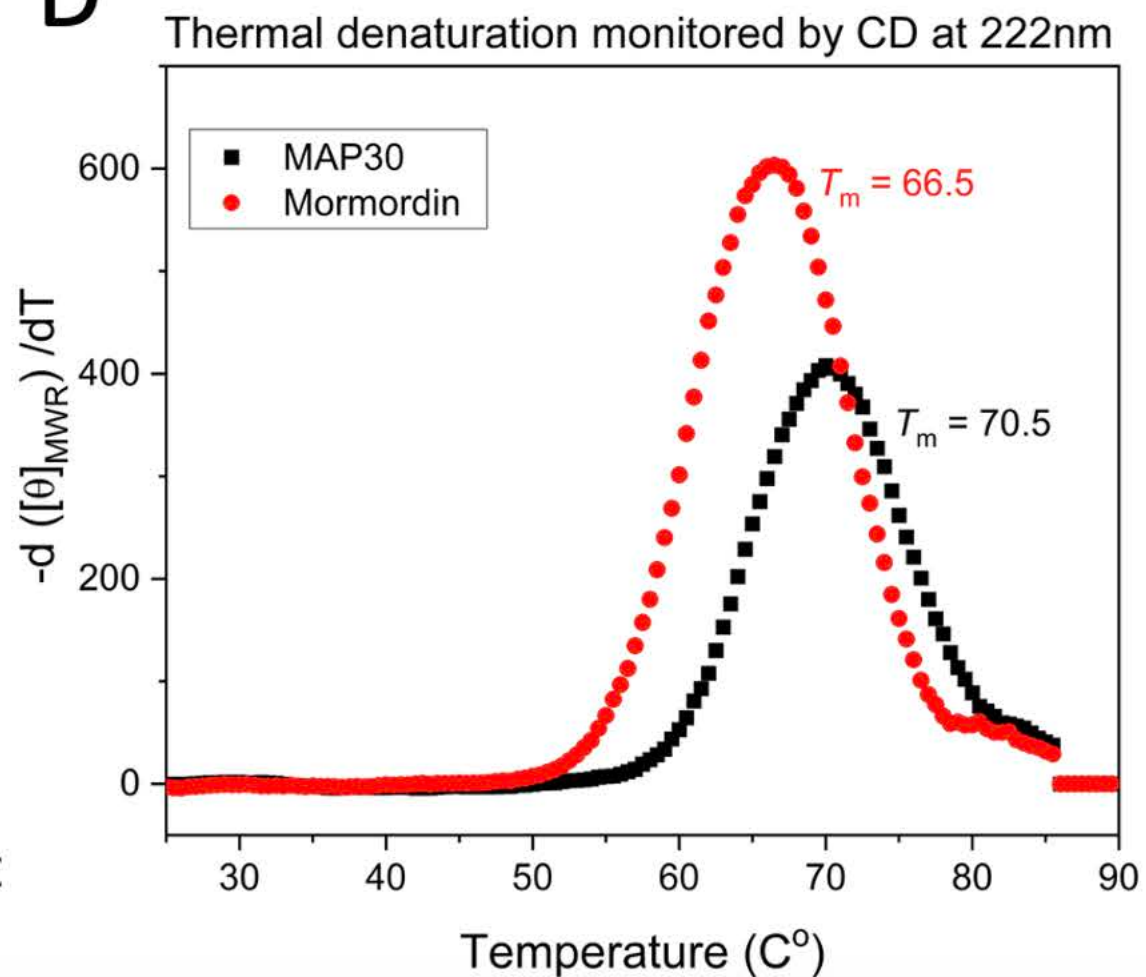

Supplement: S1 Fig — (A) SDS-PAGE of MAP30 and Momordin, with and without a C-terminally appended Tat cell penetration peptide, after the final step of column chromatography purification. Standards (Lanes 1 and 6), MAP30 (Lanes 2, 3, 4, and 7), MAP30-Tat (Lane 8), MAP30.Y70A (Lanes 9–12, column fractions), MAP30.K171A, K215A (Lanes 13–16, column fractions), Momordin (Lanes 17 and 18), Momordin-Tat (Lanes 19–22, column fractions). (B) Sedimentation equilibrium analytical ultracentrifugation of MAP30 showing that the protein is monomeric with no tendency for self-association and with a mass corresponding to that predicted from sequence (Fig 1C). (C) Far-UV circular dichroic spectra of MAP30 and Momordin. (D) Thermal denaturation of MAP30 and Momordin monitored by helix–coil transitions at 222 nm. The denaturation of both proteins was irreversible. (PDF) [file pone.0286370.s001.pdf]

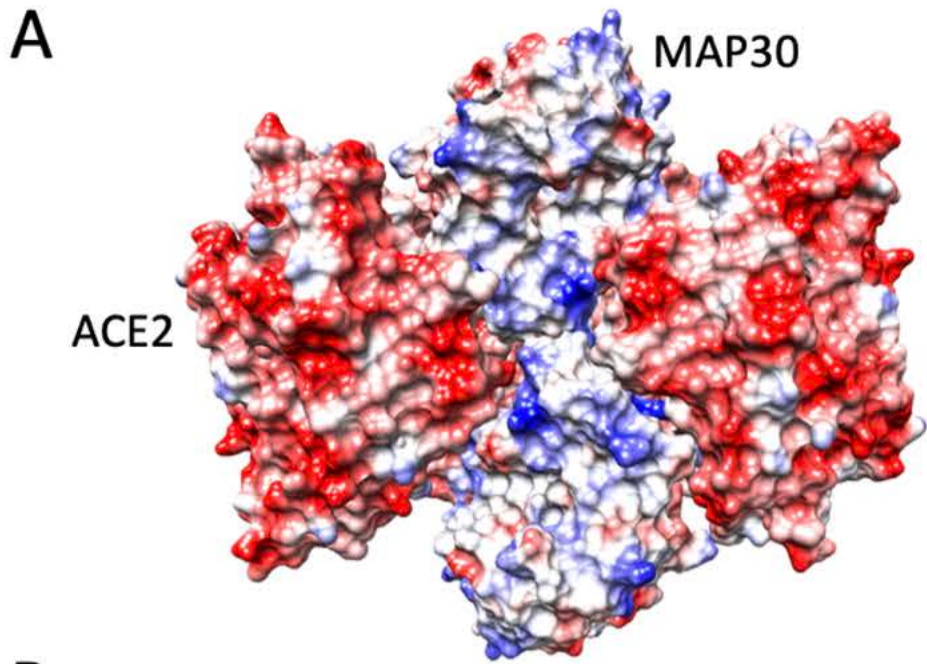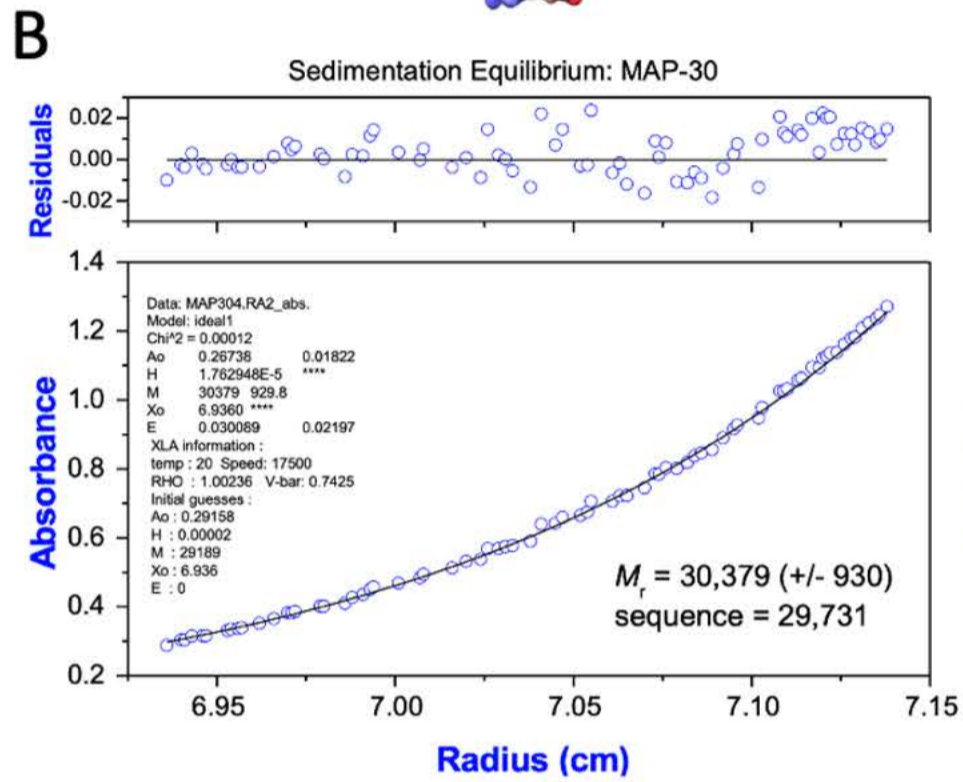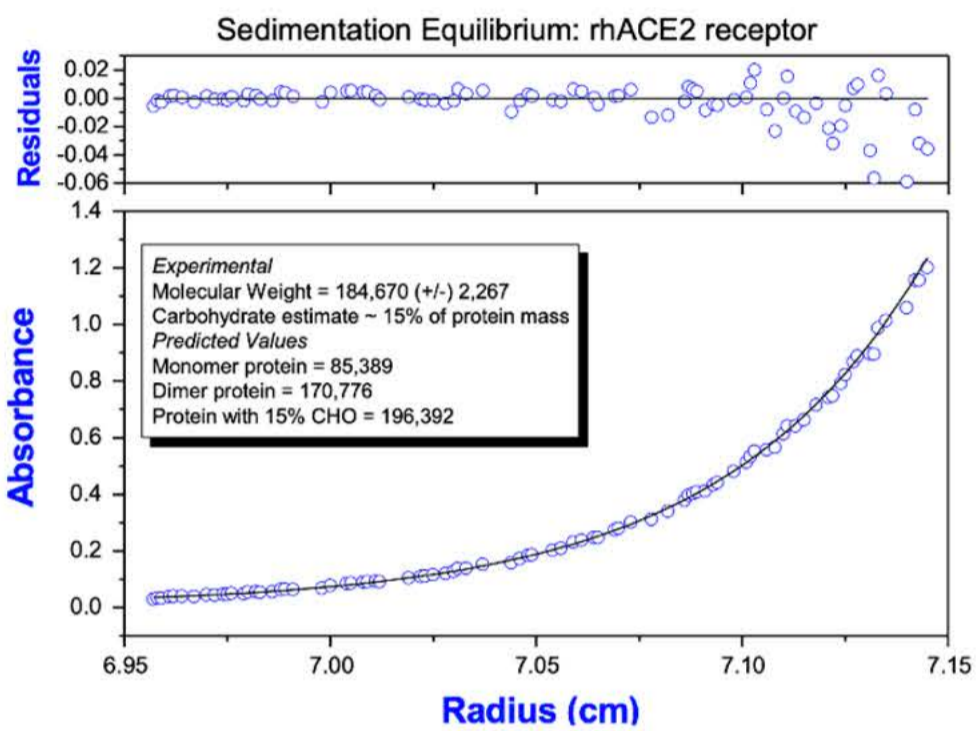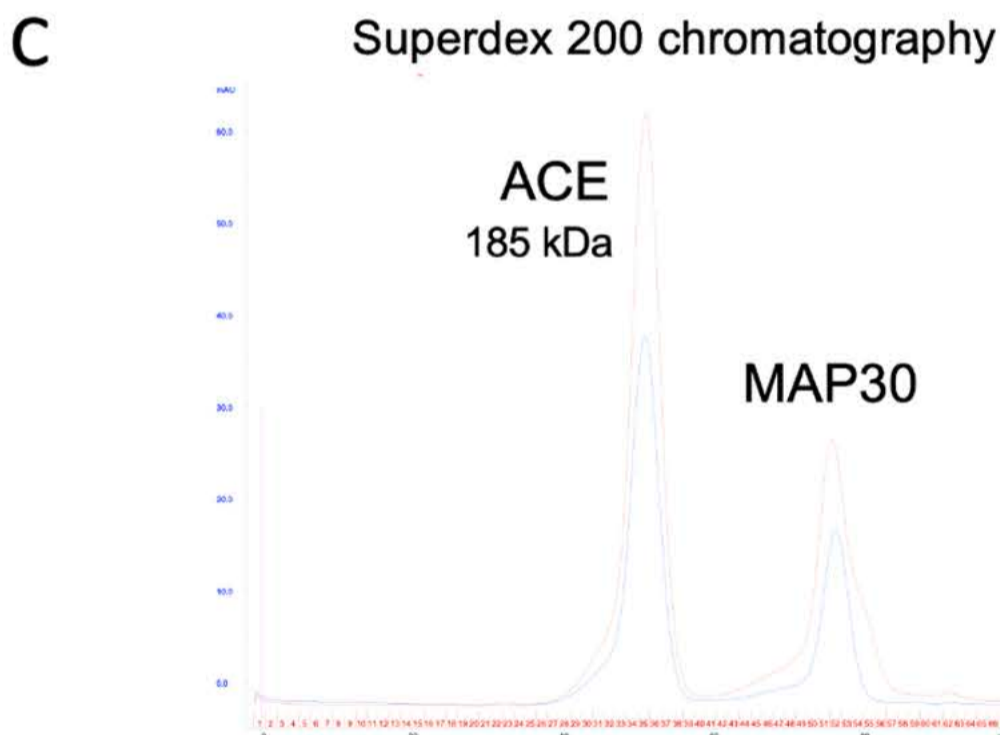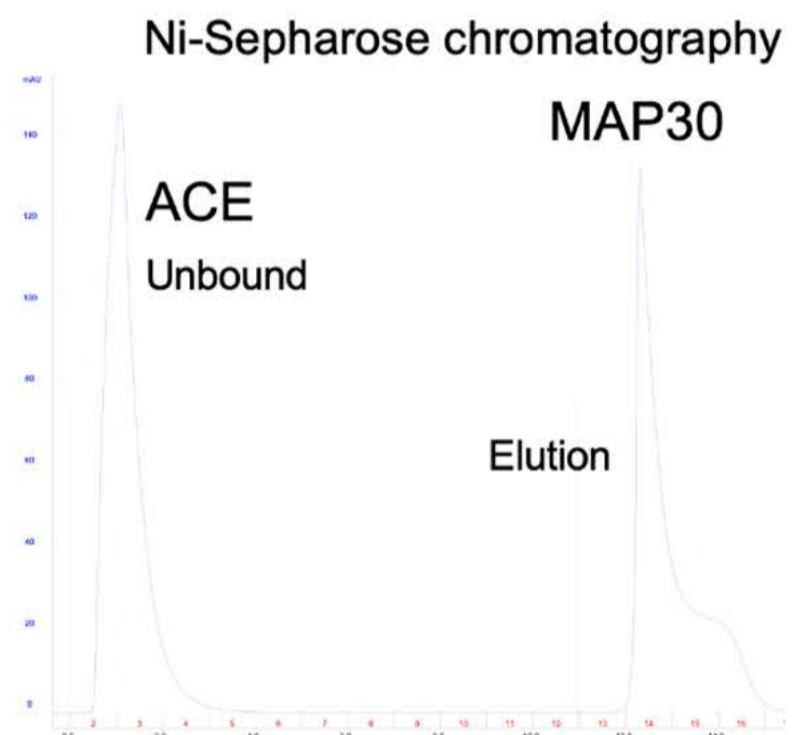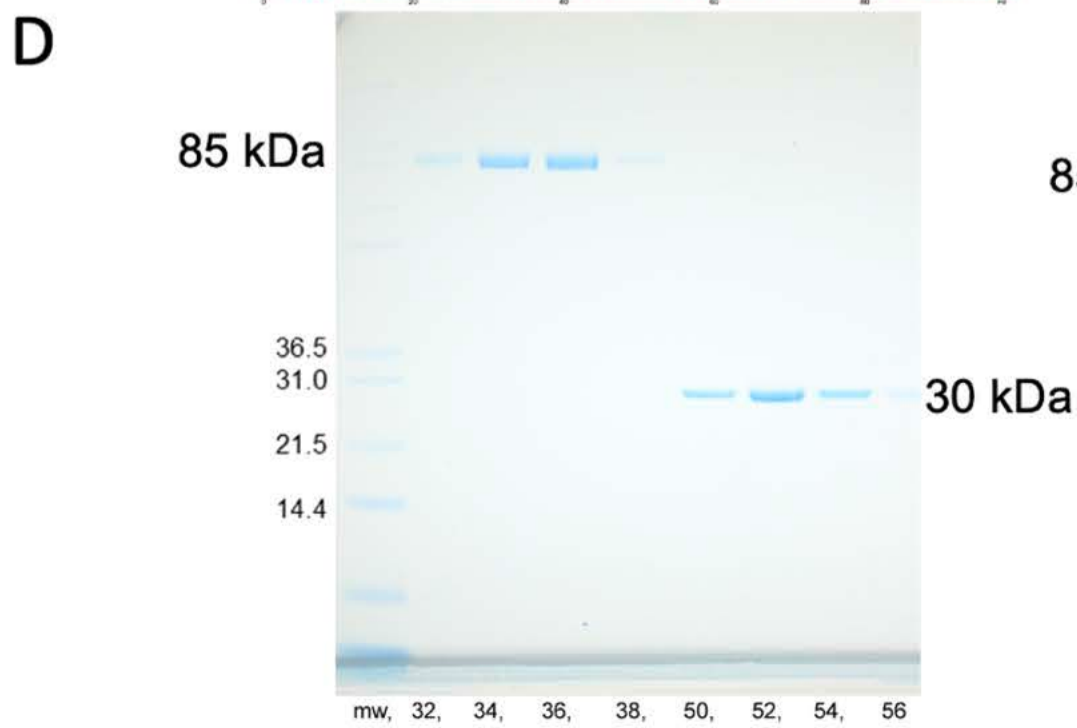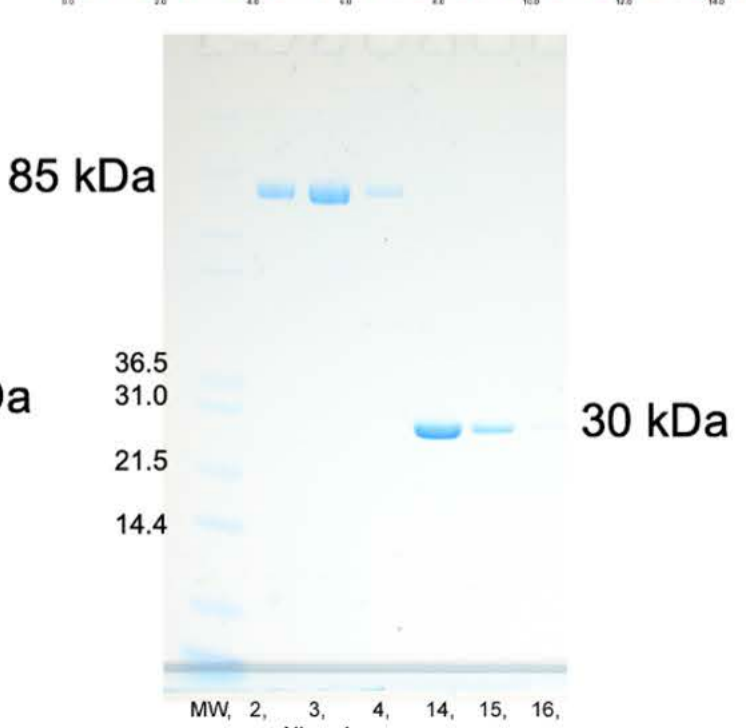

Supplement: S2 Fig — (A) Model of how MAP30 might engage ACE2. Two copies of MAP30 (PDB: 1D8V) fitted onto the ACE2 dimer (PDB: 6M18). Top view (left), and side view (right). Note the apparent shape and charge complementarity. (B) Sedimentation equilibrium analysis of MAP30 (left), and rhACE2 (APN01) (right). MAP30 is monomeric and ACE2 is dimeric. (C) Assay for interaction between MAP30 and soluble rhACE2. Separation of MAP30 and ACE2 by Superdex 200 (left), and Ni-Sepharose (right), chromatography. (D) Analysis by SDS-PAGE of fractions from Superdex 200 (left), and Ni-Sepharose (right), chromatography. There is no evidence of an interaction between MAP30 and ACE2. (PDF) [file pone.0286370.s002.pdf]

**A**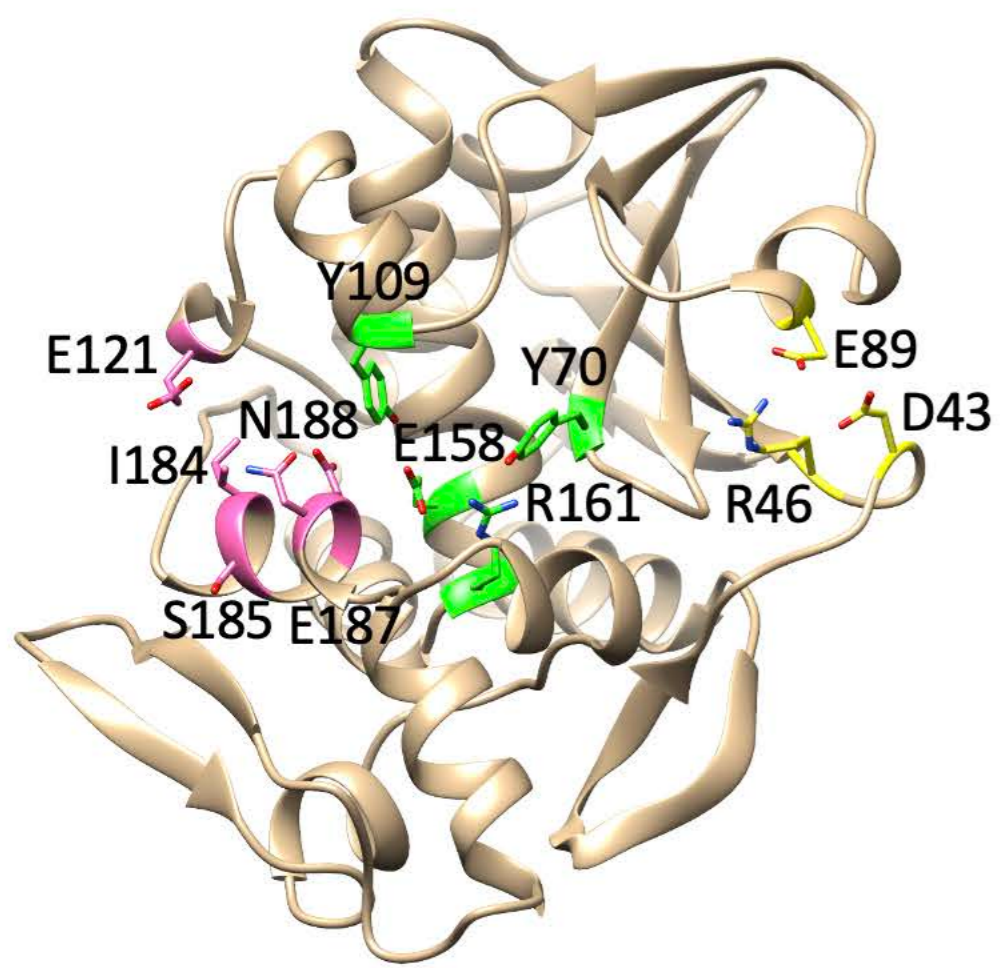

MAP30 (1D8V)

**B**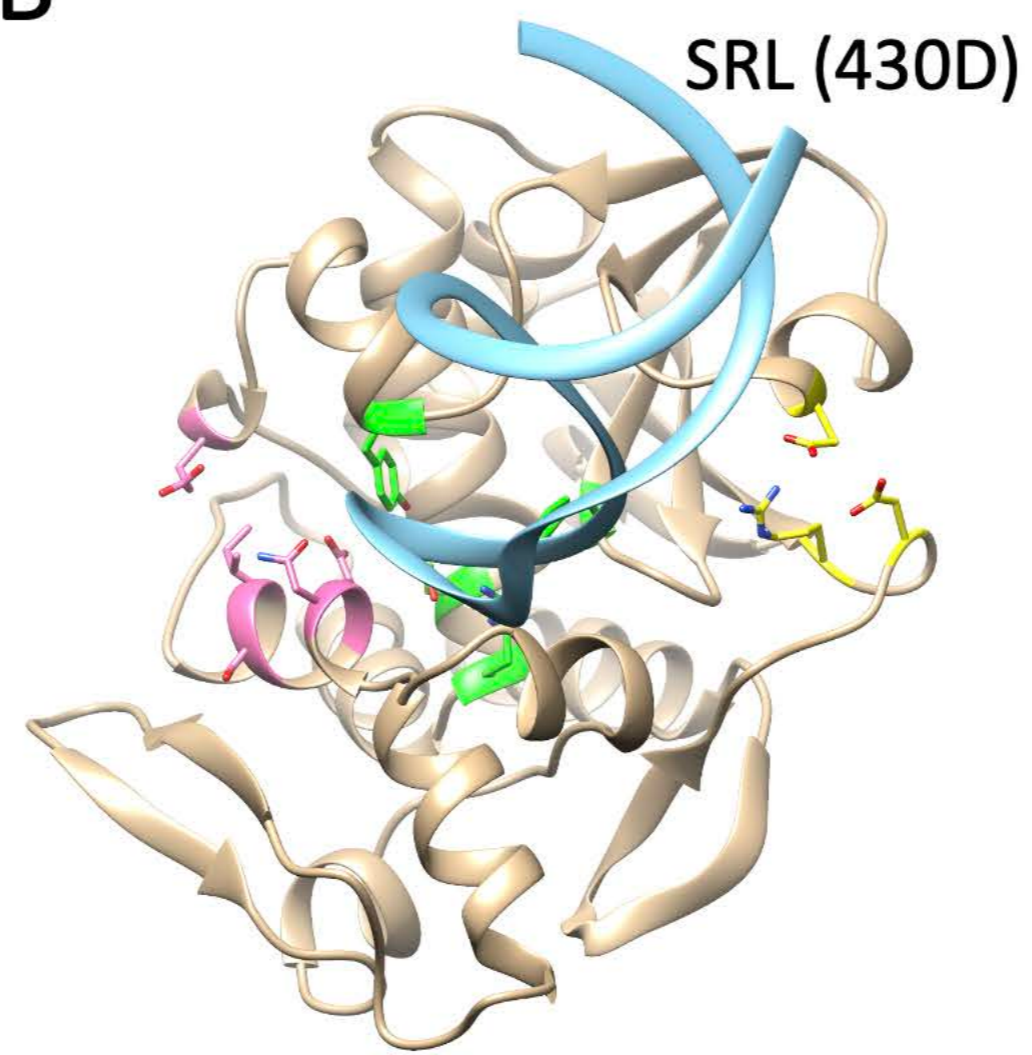

MAP30 (1D8V)

**C**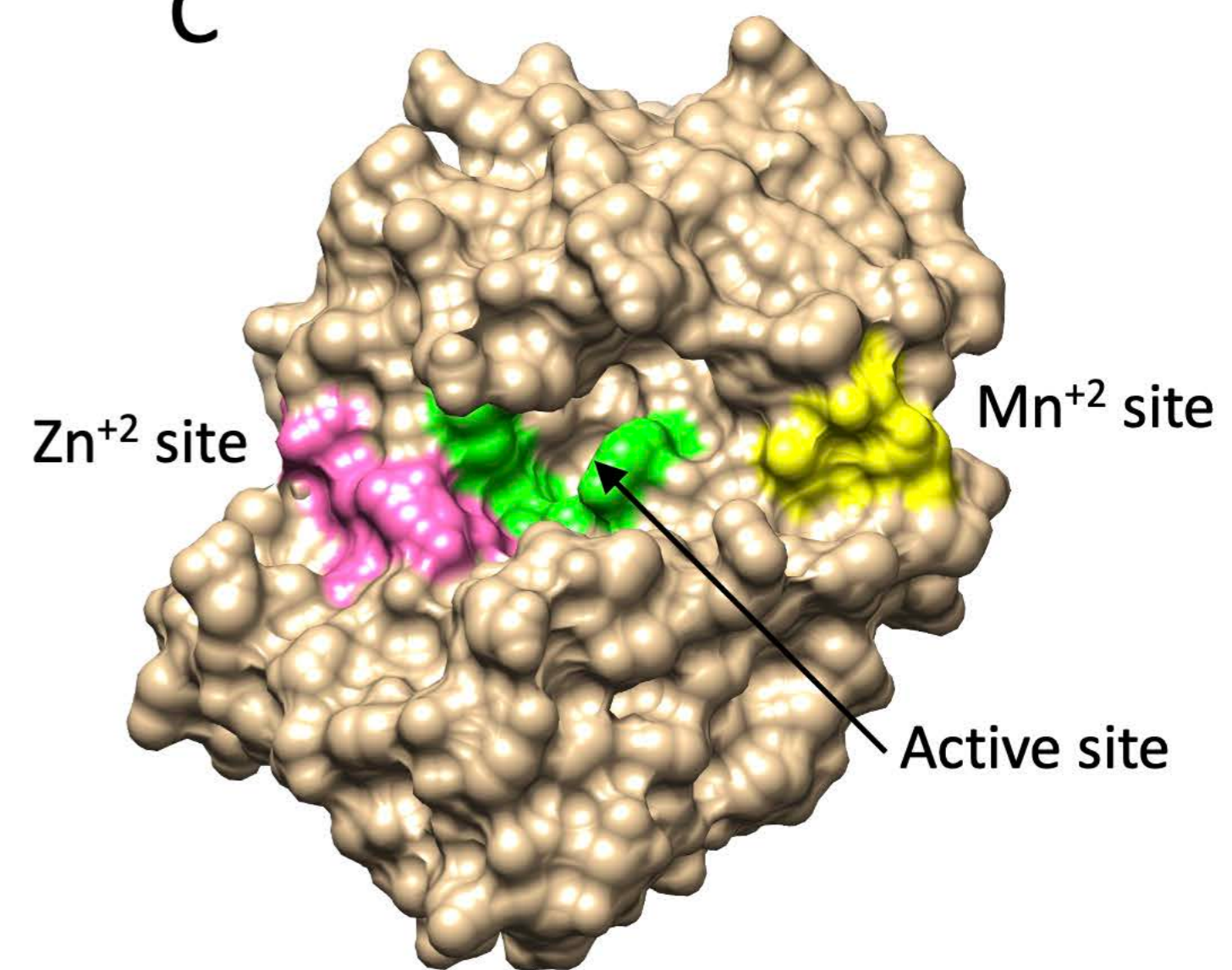**D**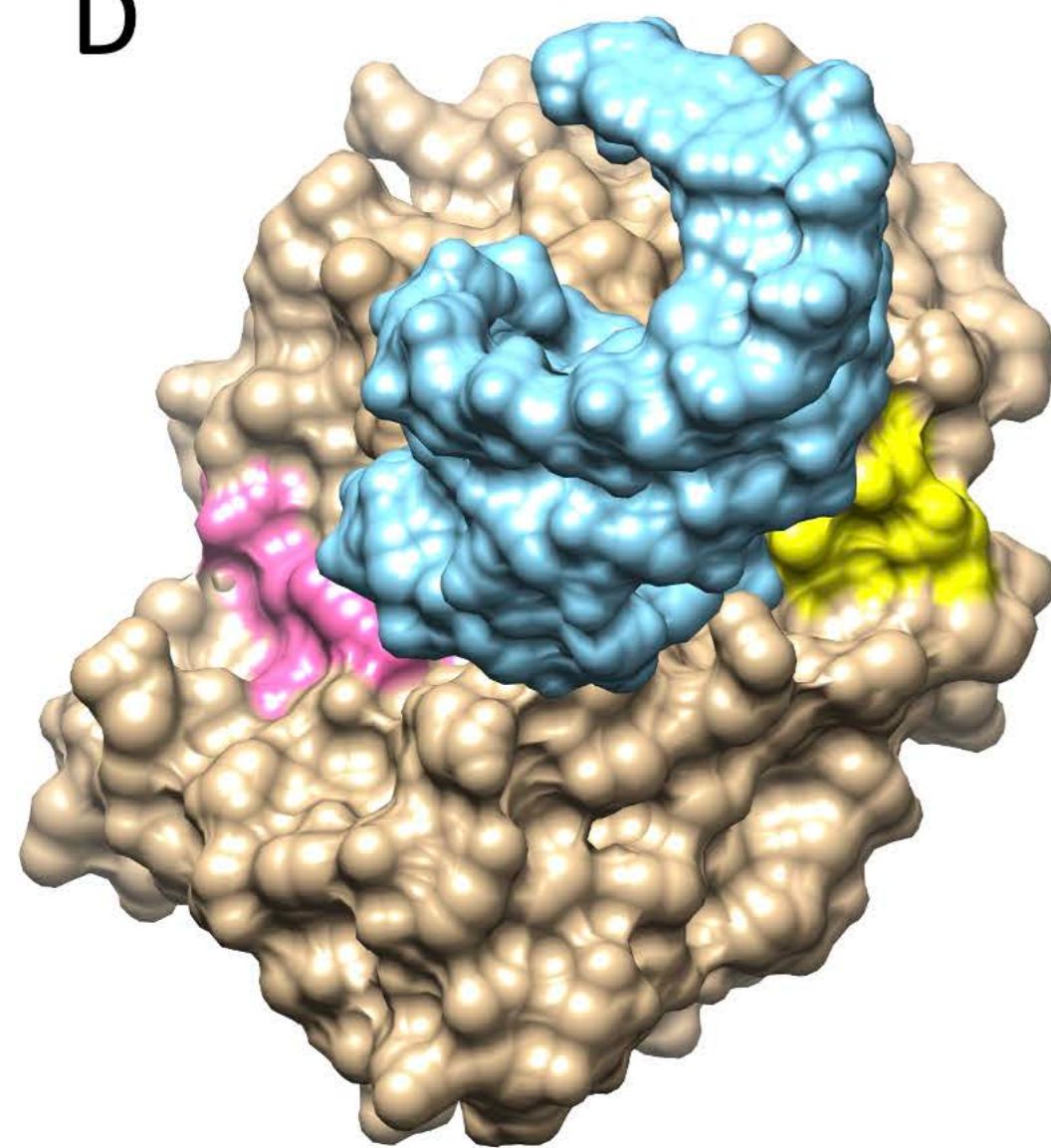**E**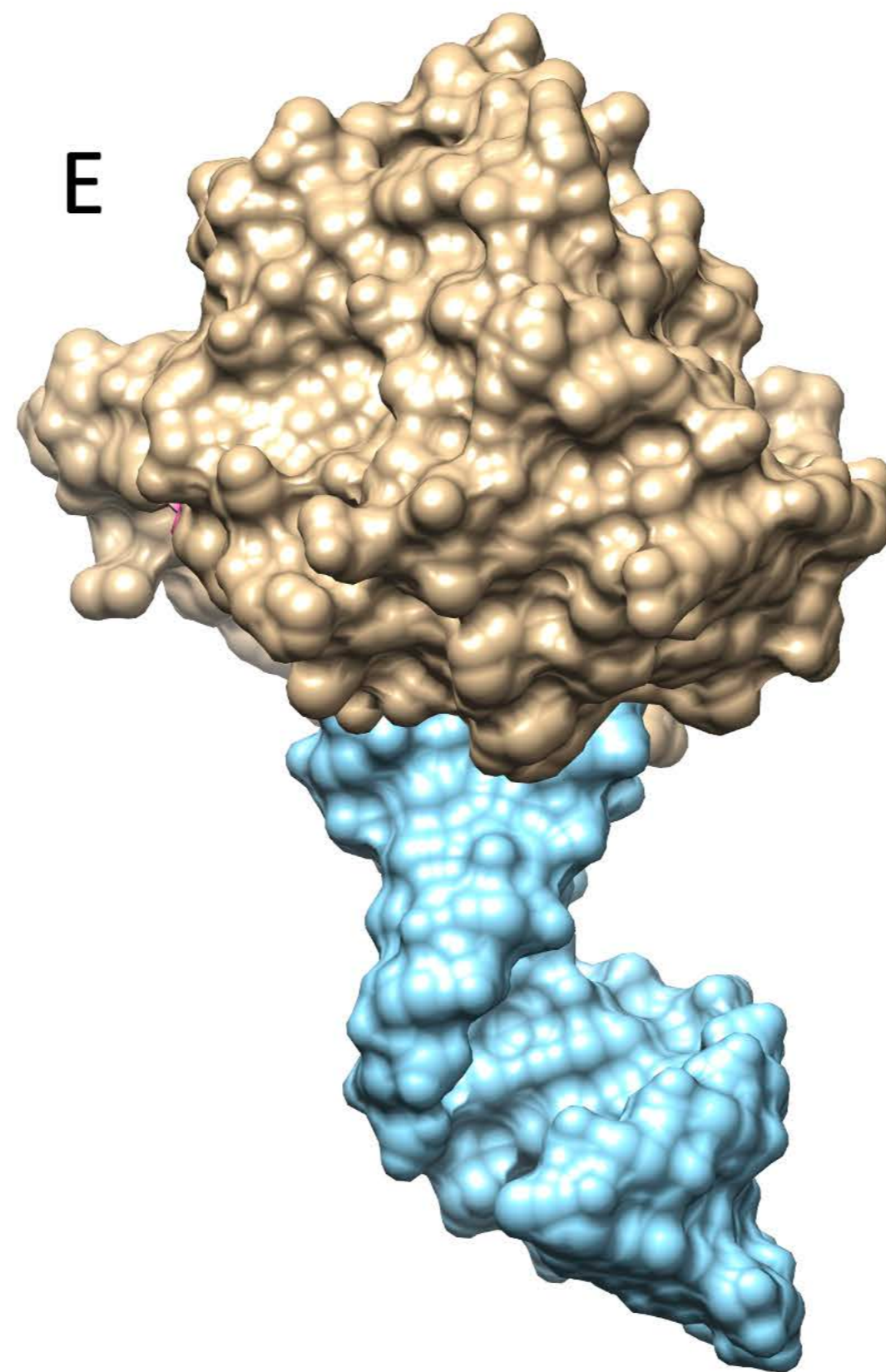**F**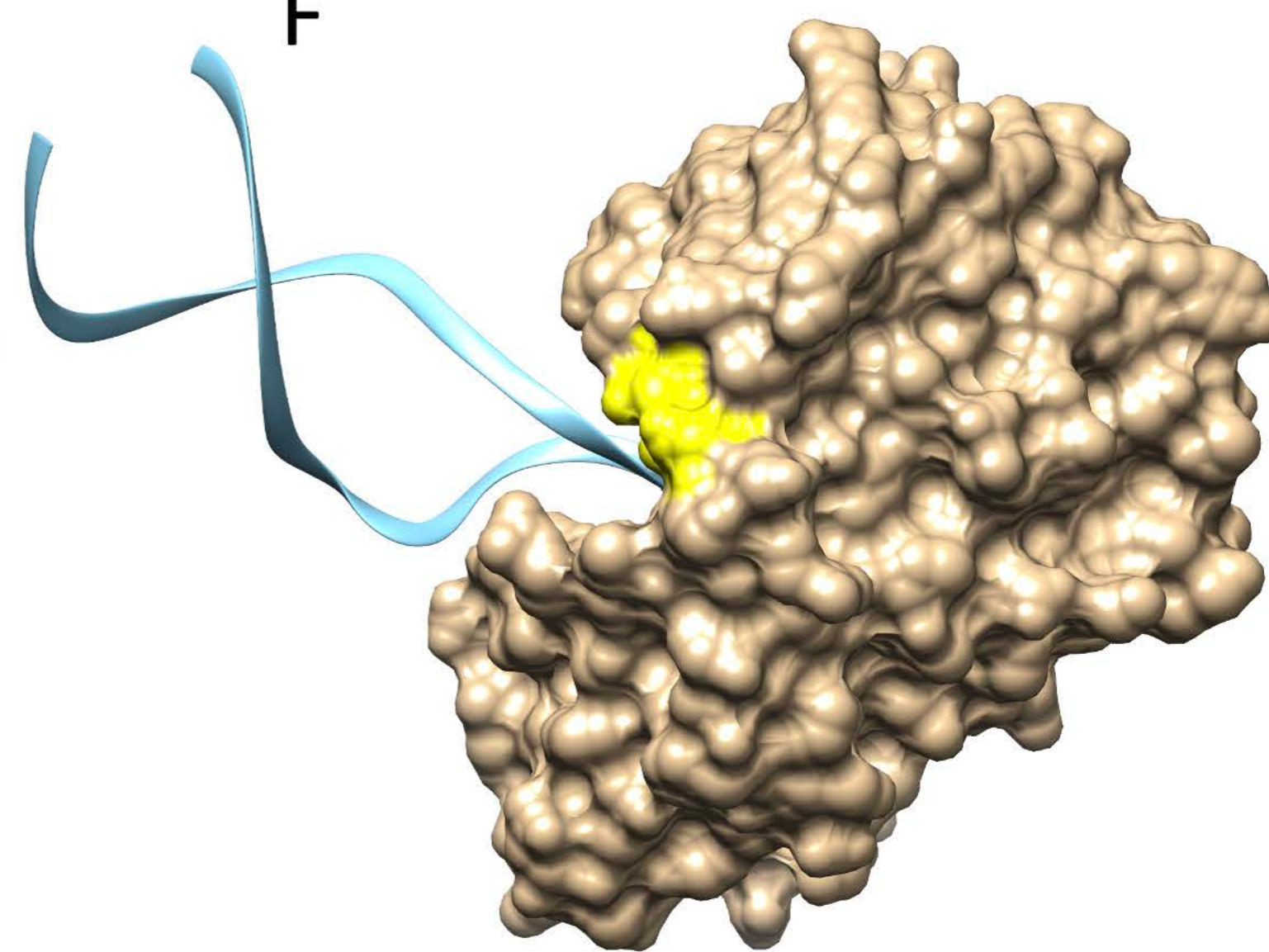

Supplement: S3 Fig — (A) Ribbon diagram of MAP30 (PDB: 1D8V) with the residues in the active site colored green, and those involved in Mn+2 and Zn+2 binding colored yellow and pink, respectively. (B) Ribbon diagram of MAP30 with the SRL (PDB: 430D) modeled to align the adenine in the GAGA motif to overlap with the bound adenine in Momordin (PDB: 1AHA) and to avoid clashes with MAP30 (C). (D-F) Orthogonal views of the MAP30-SRL complex model. (PDF) [file pone.0286370.s003.pdf]

A

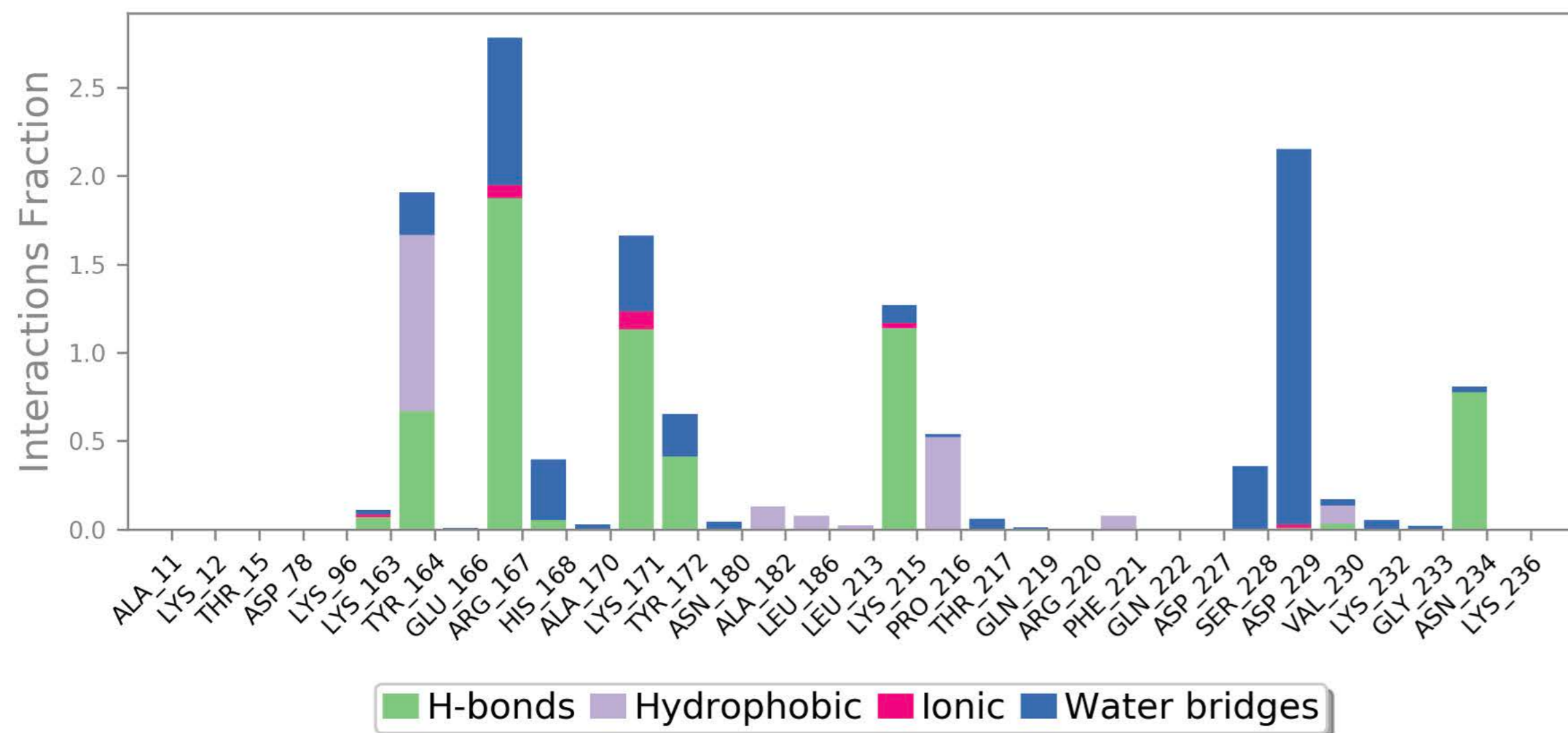

B

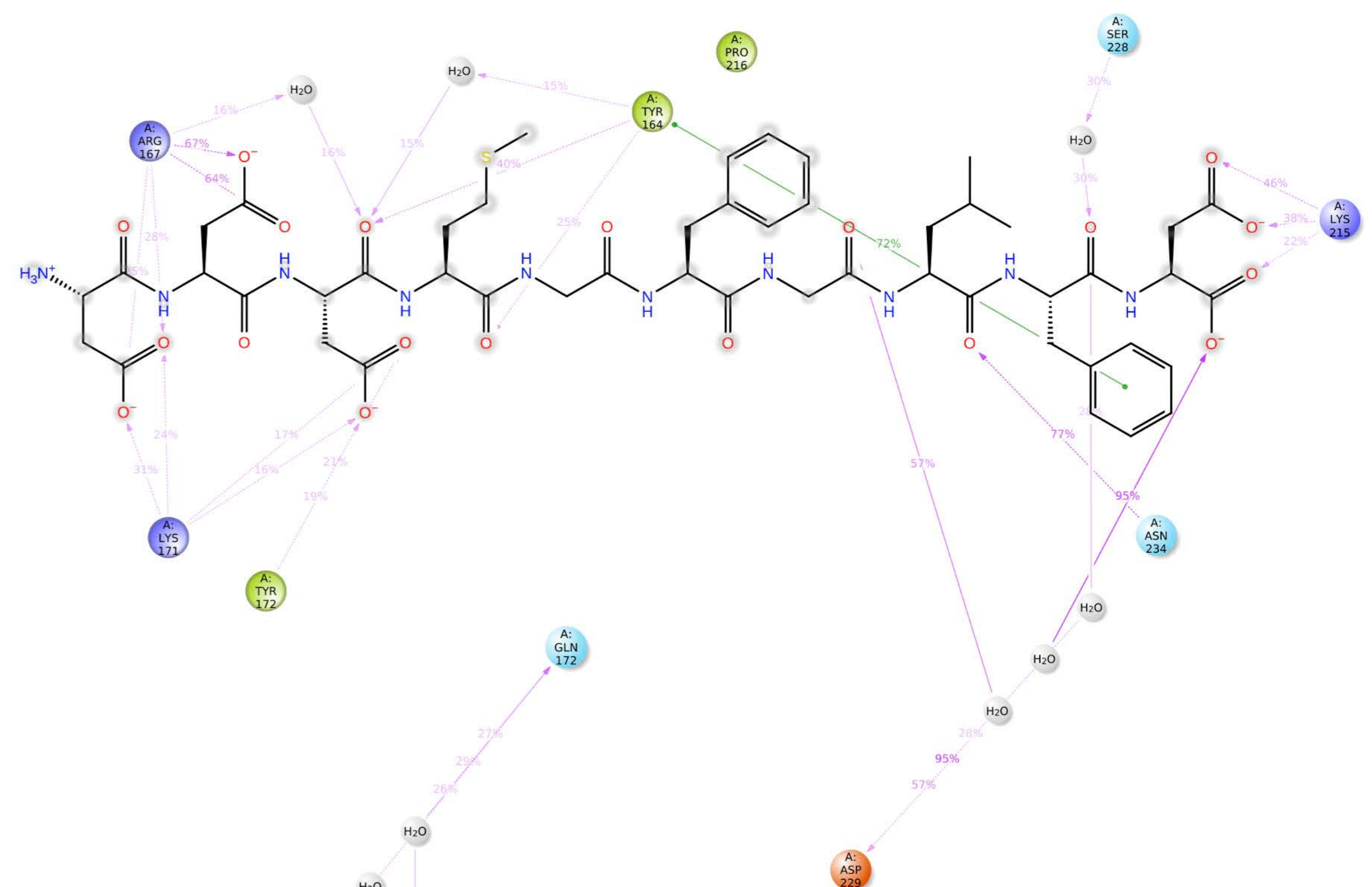

C

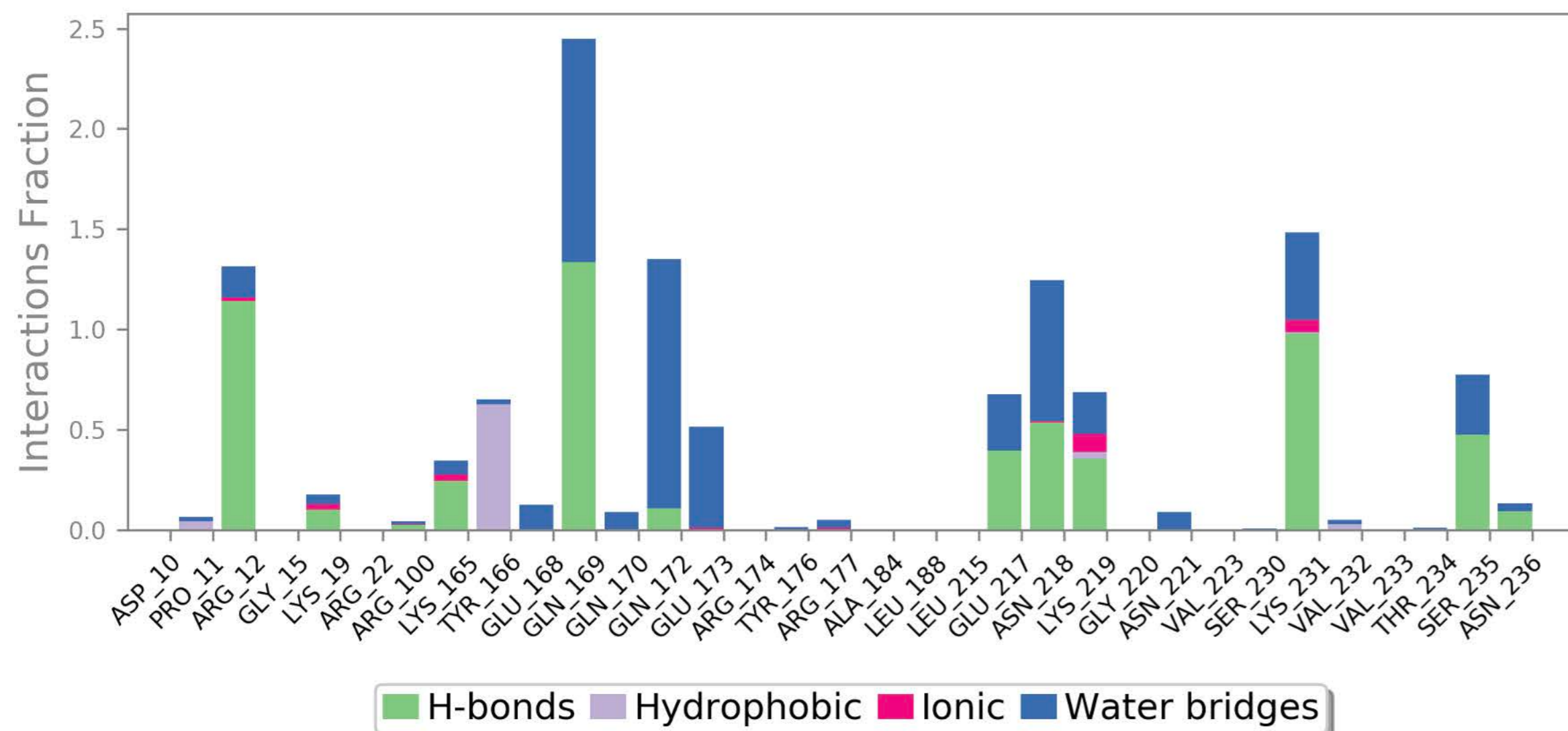

D

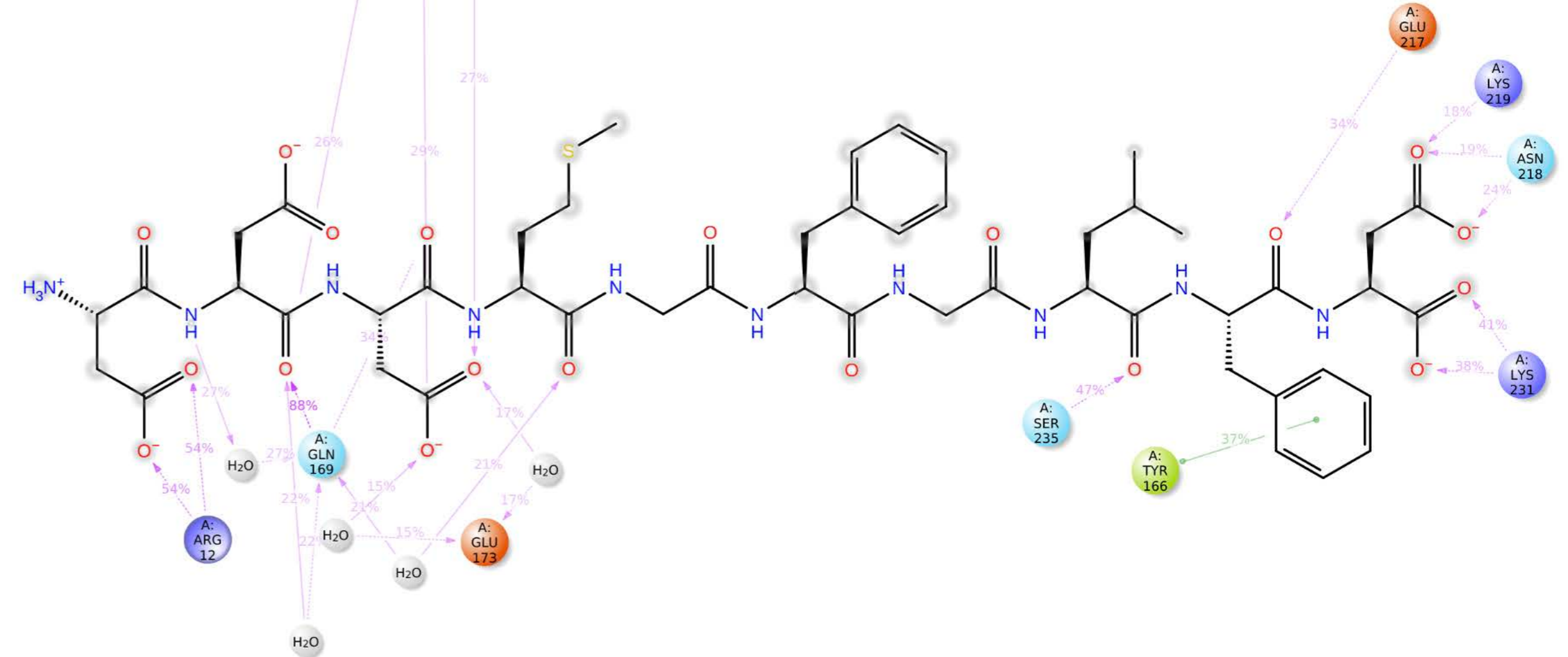

Supplement: S4 Fig — (A and C) Interaction fractions of the peptide with MAP30 and Momordin residues, respectively. (B and D) Schematics of peptide atom interactions with MAP30 and Momordin residues, respectively. Note the large contribution of the hydrophobic interaction of F10 in the peptide with Y164 in MAP30 (occurring 72% of the time during the 250 ns simulation) and to a lesser extent with Y166 in Momordin (37%). Note also, the charge interactions of D2, D3, and D4 in the N-terminal region of the peptide with R167 and K171, and of D11 and the C-terminus of the peptide with K215, in MAP30. Similar interactions occur between the C-terminal region of the peptide, but not the N-terminal region, and Momordin. (PDF) [file pone.0286370.s004.pdf]

A

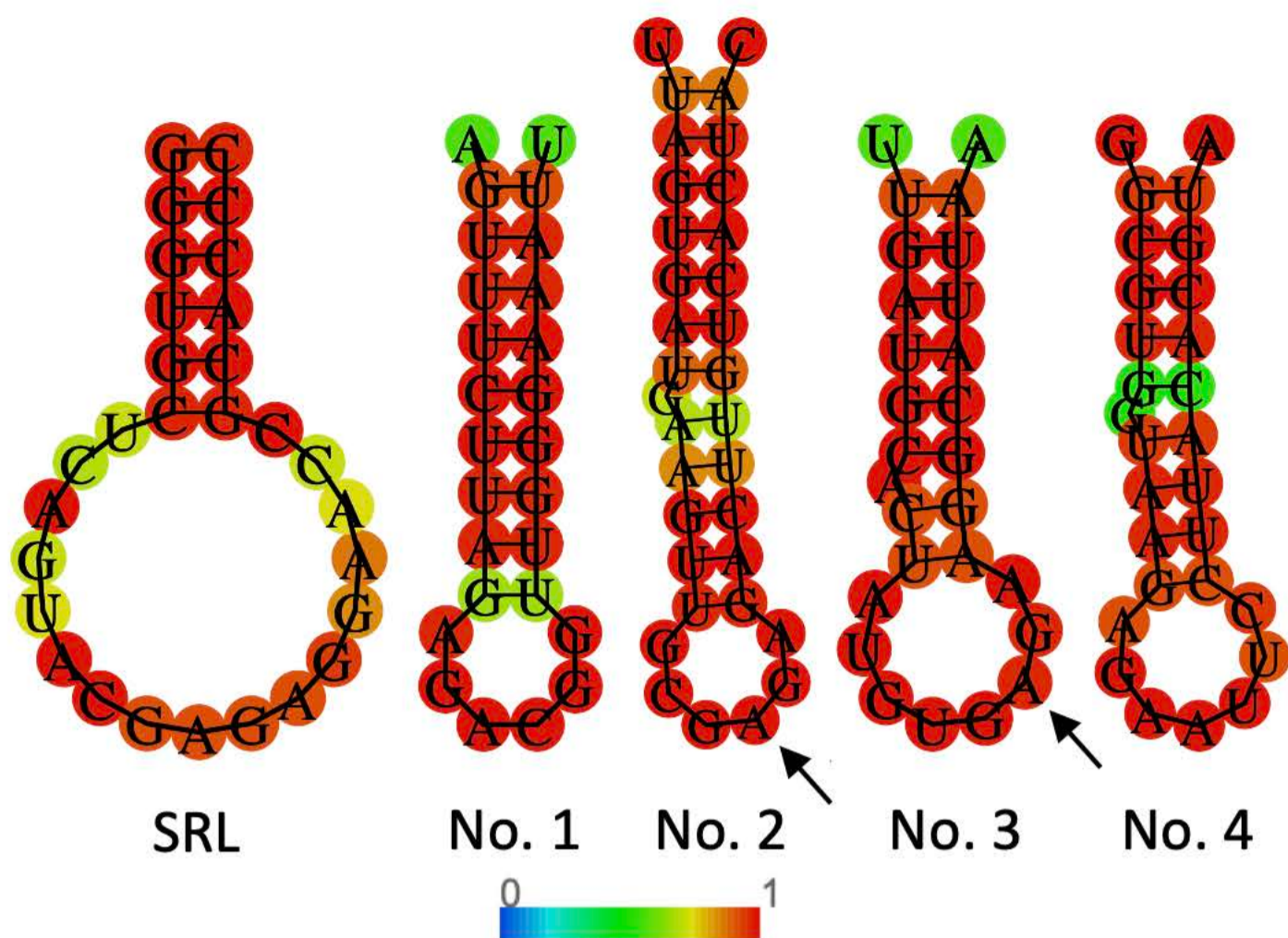

B

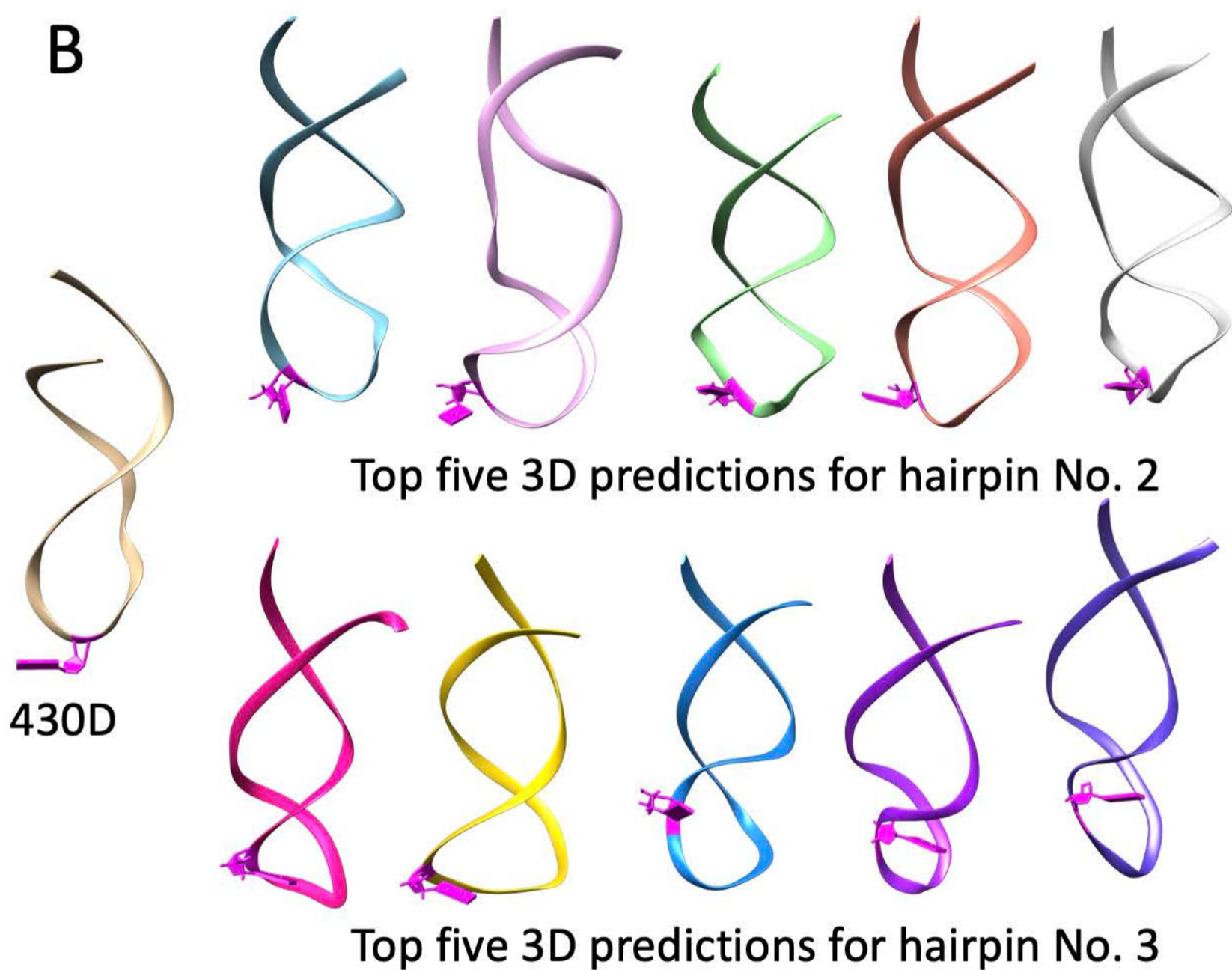

C

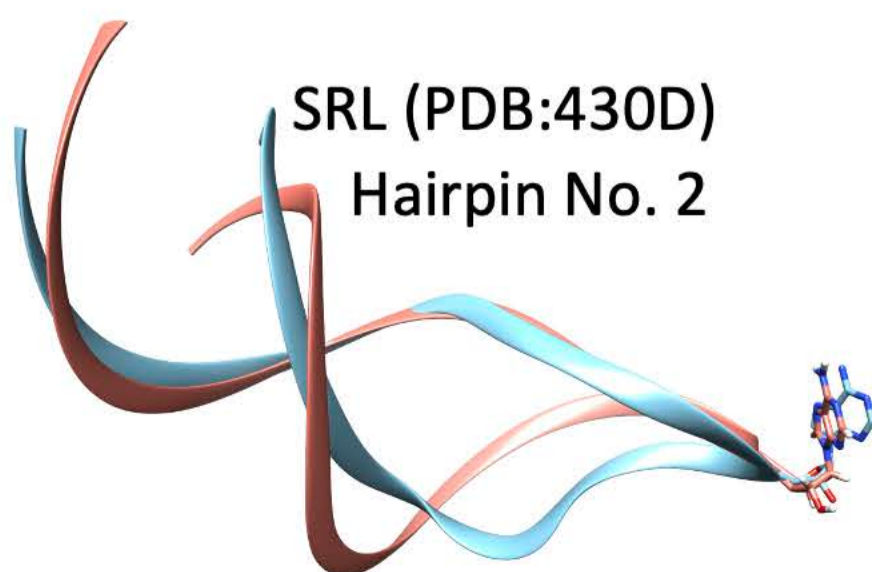

Supplement: S5 Fig — (A) 2D structure predictions for the SRL and the four stem-loop structures in the SARS-CoV-2 genome with the GAGA RIP recognition motif located in the loop. In stem-loops No. 2 and No. 3, the adenine in the GAGA motif where depurination might occur is in a similar position distal to the stem (indicated by an arrow). (B) Ribbon diagrams of (left) the SRL X-ray crystal structure (PDB: 430D), and (right) the top five 3D structure predictions for stem-loops No. 2 and No. 3 in (A). The adenine in the GAGA loop where depurination may occur is highlighted in magenta in each case. (C) Alignment of predicted Hairpin No. 2 with the SRL, with the respective first adenine bases in the common CGAGAG motif shown as sticks colored by heteroatom. The bases are extrahelical and essentially superimposable. (PDF) [file pone.0286370.s005.pdf]
