# Supplementary material for: The ribosome-inactivating proteins MAP30 and Momordin inhibit SARS-CoV-2
Source: PLoS One. 2023 Jun 29;18(6):e0286370. doi: 10.1371/journal.pone.0286370 (PMC10310010; doi:10.1371/journal.pone.0286370)
Supplement: S1 Table — (PDF) [file pone.0286370.s006.pdf]

S1 Table. MAP30 and Momordin Mn^{+2} binding sites

	PDB	Mn^{+2} site[1]	
**MAP30[2]**	1D8V	D43	E89
**MAP30**	1CF5	D43	E89
**Momordin**	1MOM	-	-
**Saporin**	1QI7	E48	E98
**Shiga toxin**	1DM0	D47	-
**Pokeweed antiviral protein**	1D6A	-	D100
**Trichosanthin**	2JDL	-	E90
**Ricin**	1RTC	-	E99

[1] For simplicity, only the Mn^{+2} site is included here, the Zn^{+2} sites are not. The Zn^{+2} sites include residues such as Ile, Ser, and Gln, not typical ion-coordinating residues such as Cys, His, Asp, and Glu. Backbone amide chemical shifts are very sensitive to even minor local perturbations and not indicative of solely direct interactions. These shifts are simply qualitative indicators of the approximate area where a ligand interacts (A. Bax, personal communication).

[2] The original structure for MAP30 (1D8V) which described the Mn^{+2} site was determined by NMR. The other structures, including MAP30 (1CF5) are X-ray crystal structures.
