# Supplementary material for: The ribosome-inactivating proteins MAP30 and Momordin inhibit SARS-CoV-2
Source: PLoS One. 2023 Jun 29;18(6):e0286370. doi: 10.1371/journal.pone.0286370 (PMC10310010; doi:10.1371/journal.pone.0286370)
Supplement: S2 Table — (PDF) [file pone.0286370.s007.pdf]

S2 Table. Binding parameters of c11-P peptide

	Complex	Area (Å^2)	ΔG (kcal/mol)[1]	p-value	N_{HB}	N_{SB}
**Trichosanthin**	2JDL	556	-3.8	0.657	9	4
**MAP30**[2]	1CF5 + 2JDL-peptide	577	-5.6	0.421	6	6
**Momordin**[3]	1AHA + 2JDL-peptide	590	-6.6	0.383	5	5

[1] All three complexes, including 2JDL, were subjected to one round of energy minimization (fastest descent and conjugate gradient) with UCSF Chimera prior to estimation of their free energy of binding with PDBePISA (as free energy of solvation, which does not include salt bridges or hydrogen bonds). The numbers of hydrogen bonds (N_{HB}) and salt bridges (N_{SB}) are shown separately.

[2] Interaction of the c11-P peptide with MAP30 was modeled by alignment of 2JDL to 1CF5, removal of the TCS chain, followed by energy minimization.

[3] Interaction of c11-P peptide with Momordin was modeled as above.
