# Supplementary material for: The ribosome-inactivating proteins MAP30 and Momordin inhibit SARS-CoV-2
Source: PLoS One. 2023 Jun 29;18(6):e0286370. doi: 10.1371/journal.pone.0286370 (PMC10310010; doi:10.1371/journal.pone.0286370)
Supplement: S3 Table — (PDF) [file pone.0286370.s008.pdf]

S3 Table. Predicted RNA hairpin folding parameters

	**Number of nucleotides**	**Position in genome**	**Coding region**	**Free energy[1] (kcal/mol)**	**Frequency[2] (%)**	**Diversity[3]**
**SRL**	29	-	-	-9.2	41.7	2.1
**No. 1[4]**	27	2183-2209	NSP2	-5.58	33.18	1.48
**No. 2[5]**	33	7659-7691	NSP3	-11.34	49.31	1.87
**No. 3[6]**	27	17177-17203	NSP13	-6.49	45.00	1.12
**No. 4[7]**	28	17716-17743	NSP13	-8.57	39.68	1.78

[1] Free energy of the ensemble as determined with RNAfold.
[2] Frequency in the ensemble, determined as above.
[3] Diversity of the ensemble, determined as above.
[4] GAGUUUCUUAG**A**GACGGUUGGGAAAUU
[5] UUAGUGAUGAAGUUGCG**A**GAGACUUGUCACUAC
[6] UUGAUGCACUAUGUG**A**GAAGGCAUUAA
[7] GGCGUGGUAAG**A**GAAUUCCUUACACGUA
