# Supplementary material for: The ribosome-inactivating proteins MAP30 and Momordin inhibit SARS-CoV-2
Source: PLoS One. 2023 Jun 29;18(6):e0286370. doi: 10.1371/journal.pone.0286370 (PMC10310010; doi:10.1371/journal.pone.0286370)
Supplement: Attachment — Submitted filename: Response to Reviewers.docx [file pone.0286370.s012.docx]

**Response to Reviewers:**

Reviewer #1: The authors have provided responses to my first concern about the use of A549 cells from a retrospective (historical) manner. They have also included now in the revised manuscript more molecular simulation experiments which were placed in the Supplementary section. The authors also provided explanations on concerns about data discrepancy both in the responses to my concern and to another reviewer's questions. Overall, the paper is now improved in terms of its scientific basis and presentation.

I still recommend that the authors make the justification on the use of A549 clearer in the manuscript, so as to help the audience understand the seemingly contradictory results in A549 and Vero experiments.

The Reviewer has requested that we justify our choice of assay, and to explain our results more clearly.

The assays were performed with both A549 and Vero cells. Both cell lines were being used at Southern Research to assay potential SARS-CoV-2 inhibitors. The A549 NLRV assay was expected to provide information about replication inhibition. The Vero CPE assay was expected to provide information on overall viral inhibition, due to both extracellular (entry) and intracellular (replication) inhibition.

The results obtained with the A549 and Vero cell assays are not contradictory. Our data show a ten-fold difference between the 50% viral-inhibitory and cell-toxicity concentrations with the A549 cells and no difference with the Vero cells. Why the Vero cells did not “work” in this situation is something that remains to be determined. Our gel-filtration, affinity chromatography, and analytical ultracentrifugation results suggest that viral entry inhibition by blocking of the ACE2 receptor was not a factor. That the toxicity concentration for the A549 and Vero cells was very similar suggests that MAP30 entered both cells equally, and that something involved in viral replication is different in the Vero cells.

Our personal communications regarding this matter with Dr. Paige Vinson, Director of High-Throughput Screening at Southern Research, and with Drs. Timothy Sheahan and Ralph Baric at the University of North Carolina (the Baric lab is expert in this field and had provided both the A549 cells expressing ACE-2 and the USA_WA1/2020 nanoluciferase reporter construct to Southern Research to assay potential SARS-CoV-2 replication inhibitors), provided no explanation for why the Vero assay did not work. It may just be that human lung cells are more appropriate than monkey kidney cells in this case. Other cells, such as osteoclasts, keratinocytes, retinal ganglia, or follicular cells, may also prove unsuitable for assaying SARS-CoV-2 replication inhibition. A549 cells are commonly used to model the alveolar Type II pulmonary epithelium. They have been used to study various conditions including cancer, allergies, asthma, and respiratory infections. The A549 cell line has been approved by the FDA for use in a variety of applications, including in vivo testing of drugs and other therapeutics for use in clinical trials. We therefore think that the A549 assay data are relevant. As with many potential therapeutics, it is the differential between on and off-target effects that is of interest. Clearly, RIPs are not yet ready to be used as therapeutics, but our results demonstrate viral inhibition in a relevant cell type. When the molecular details of what is going on in the Vero cells is known, viral inhibition by RIPs will be better understood.

To provide more information to readers on how the cells were chosen we have added the following text at line 331.

“Both the reporter virus and the cells had been provided to Southern Research by the laboratory of Ralph Baric (UNC) specifically to assay potential SARS-CoV-2 replication inhibitors. A549 cells are commonly used to model the alveolar Type II pulmonary epithelium. They have been used to study various conditions including cancer, allergies, asthma, and respiratory infections.”

To clarify the results from the Vero assays we have added the following text at line 568.

“These observations suggest that SARS-CoV-2 inhibition by RIPs may be cell line specific and that perhaps in Vero cells some factor involved in viral replication is different from that A549 cells.”
